# Disentangling the Neural Basis of Cognitive Behavioral Therapy in Psychiatric Disorders: A Focus on Depression

**DOI:** 10.3390/brainsci8080150

**Published:** 2018-08-09

**Authors:** Moussa A. Chalah, Samar S. Ayache

**Affiliations:** 1 EA 4391 Excitabilité Nerveuse et Thérapeutique, Université Paris-Est, 94010 Créteil, France; samarayache@gmail.com; 2 Service de Physiologie-Explorations Fonctionnelles, Hôpital Henri Mondor, Assistance Publique—Hôpitaux de Paris, 94010 Créteil, France; 3 Neurology Division, Lebanese American University Medical Center-Rizk Hospital (LAUMC-RH), Beirut 1100, Lebanon

**Keywords:** depression, MDD, neuroimaging, MRI, cognitive behavioral therapy, CBT, cognitive therapy, behavioral therapy

## Abstract

**Background:** Major depressive disorder (MDD) stands among the most frequent psychiatric disorders. Cognitive behavioral therapy (CBT) has been shown to be effective for treating depression, yet its neural mechanisms of action are not well elucidated. The objective of this work is to assess the available neuroimaging studies exploring CBT’s effects in adult patients with MDD. **Methods:** Computerized databases were consulted till April 2018 and a research was conducted according to PRISMA guidelines in order to identify original research articles published at any time in English and French languages on this topic. **Results:** Seventeen studies were identified. Only one study was randomized comparing CBT to pharmacological interventions, and none included an effective control. Following CBT, changes occurred in cerebral areas that are part of the fronto-limbic system, namely the cingulate cortex, prefrontal cortex and amygdala-hippocampal complex. However, the pattern of activation and connectivity in these areas varied across the studies. **Conclusion:** A considerable heterogeneity exists with regard to study design, adapted CBT type and intensity, and employed neuroimaging paradigms, all of which may partly explain the difference in studies’ outcomes. The lack of randomization and effective controls in most of them makes it difficult to draw formal conclusion whether the observed effects are CBT mediated or due to spontaneous recovery. Despite the observed inconsistencies and dearth of data, CBT appears to exert its anti-depressant effects mainly by modulating the function of affective and cognitive networks devoted to emotions generation and control, respectively. This concept remains to be validated in large scale randomized controlled trials.

## 1. Introduction

Major depressive disorder (MDD) stands among the most prevalent psychiatric disorders and accounts for a considerable part of mental health disease burden in Western countries [1]. MDD has an estimated lifetime prevalence of 17% [2] and is characterized by an early onset (i.e., adolescence and young adulthood), chronicity and recurrence, higher frequency in women, and a drastic impact on quality of life, with impairment in social and occupational domains [3].

Through the past three decades, Beck has suggested a cognitive model for MDD [4,5,6,7]. In this model, prepotent maladaptive schematic representations of the self, world and future, may lead to preferential processing for schema-congruent negative self-referential information resulting in a dominance of negative thoughts, images and interpretations within the stream of consciousness that might interfere with processing of more positive, schema-incongruent information [4,5,6]. Therefore, de-activating the disorder-related maladaptive schemas and strengthening access to more reflective, realistic and adaptive modes of thought and behavior constitute the bases of the psychotherapeutic approach for MDD developed by Beck and colleagues [7].

Cognitive behavioral therapy (CBT) is a type of psychotherapy that works on cognitions and behaviors by applying several sessions over weeks in individual or group format. CBT educates individuals about how one’s thoughts and behaviors affect the perceived symptom experience [8]. It trains them to acquire coping skills aiming to modify thoughts and behaviors, and subsequently to consolidate and maintain these skills through structured practice and feedback in order to prevent symptom relapse [8].

In the context of MDD, CBT seems to be as effective as pharmacological therapies but its effects last longer [9,10,11]. Over the last decades, the advances in neuroimaging techniques made it possible to unveil several cerebral hubs that constitute neural underpinning of several neuropsychiatric diseases including MDD. A thorough understanding of CBT underlying mechanisms of action may help optimizing patients’ care and improving their quality of life. This may also help determining predictors for treatment effectiveness in this population by providing a priori estimation of outcomes and hence offering the possibility to individualize therapies.

The aim of the current work is to address CBT biological mechanisms of action in adult patients with MDD. The following section states the adapted selection criteria. This would be followed by a brief reappraisal of the neural substrates of depression. Afterwards, studies investing neural underpinnings of CBT in MDD are reviewed. Finally, a summary of the available data is provided, and some suggestions are proposed aiming to pave the way for future large-scale studies that would be helpful to increase the current understanding of this area.

## 2. Selection Criteria

Computerized databases (MEDLINE/PubMed and Scopus) were consulted, and a search was conducted independently by both coauthors according to PRISMA guidelines [12] in order to identify original research articles published at any time until 21 May 2018 in English and French languages regarding CBT mechanisms of action in MDD. Only studies that considered CBT mechanisms of action as the primary outcome and included imaging before and after CBT were considered for this review. The following key terms were used: (‘cognitive behavioral therapy’ OR ‘CBT’ OR ‘cognitive therapy’ OR ‘CT’ OR ‘behavioral therapy’ OR ‘BT’ OR ‘psychotherapy’) AND (‘depression’ OR ‘major depressive disorder’ OR ‘MDD’ OR ‘mood disorder’ OR ‘mood’) AND (‘mechanisms of action’ OR ‘underlying mechanisms’ OR ‘magnetic resonance imaging’ OR ‘MRI’ OR ‘fMRI’ OR ‘positron emission tomography’ OR ‘PET’ OR ‘single photon emission computed tomography’ OR ‘SPECT’ or ‘spectroscopy’). The eligibility and possible inclusion of each article was assessed by both authors who screened the titles, abstracts and full texts of all references retrieved in the searches based on the predetermined selection criteria. In cases of uncertainty, both authors checked the full text of the manuscript in question to assess its eligibility, and a final decision was made regarding inclusion/exclusion.

We only included studies that have evaluated neurobiological changes due to CBT through neuroimaging techniques in adult patients with MDD. Studies that included populations others than MDD, or assessed patients other than adults (pediatric, adolescent or geriatric), or did not include post-CBT imaging, or used other types of psychotherapies, were not considered.

## 3. A Brief Overview of the Depressed Brain

Current evidence supports the involvement of several cerebral regions in the pathophysiology of depression, and in the generation of various symptoms observed in patients with MDD. The cognitive control of emotion appears to involve a network, known as the dorsal network. It includes several higher-order cortical areas such as the orbitofrontal cortex (OFC), anterior cingulate cortex (ACC), and prefrontal cortex (PFC), which also participate in higher-order cognitive functions such as working memory, reasoning, problem solving, and self-referential processing [13,14,15,16,17]. These are considered as ‘emotionally cold areas’ and are thought to underlie the cognitive deficits encountered in MDD [18]. In fact, individuals suffering from depressive disorders were found to have decreased activation in some areas of this network.

In addition, some studies have documented an increased regional activation or connectivity within a ventral limbic affective network that includes the amygdala and hippocampus (considered as ‘emotionally hot areas’ underlying the generation of negative thoughts) during emotion/cognitive processing. Such a finding may explain the generation of dysphoria (excessive negative mood) in depressed patients [13,18,19,20,21,22,23,24,25,26].

Based on these data and the abovementioned model of MDD, depression seems to entail a selective enhancement of automatic processes resulting from sustained activation of schemas and/or a selective impairment of cognitive inhibitory control over these negative contents [1,27]. Therefore, one could hypothesize that CBT may act by decreasing the activity in the amygdala-hippocampal complex (AHC) (involved in bottom-up emotion processes) and/or improving the activity of cortical areas (i.e., PFC, OFC and ACC) involved in (top-down) cognitive control of emotions [1,13,21,22]. Here, it is worth noting that, although there is a kind of agreement on the role of PFC/OFC in top-down control and the involvement of AHC in bottom-up processes, some reports suggest that ACC activity be more complex than that of AHC and PFC [1]. ACC seems to be involved in both top-down and bottom-up processing and appears to play the role of an amplifier and filter of emotional and self-referential information via its connections downward to subcortical structures (e.g., thalamus) and upward to cortical structures (e.g., PFC) [28,29].

Beside the dorsal cognitive and ventral affective networks, the default mode network (DMN) appears to play a role in MDD. Its main nodes are the medial PFC, ACC, posterior cingulate cortex (PCC), precuneus, inferior parietal cortex, and lateral temporal cortex [30]. This network exhibits high metabolic activity during internally focused tasks, including meditation, and demonstrates deactivation during performance of cognitive tasks where the attention is directed toward the external environment [18,30]. An abnormal enhancement (or a reduced deactivation) in this network’s connectivity was observed in depressed patients during the performance of goal-directed tasks and appears to be associated with depressive rumination, the process of passive and repetitive thinking about one’s negative feelings, possible causes and consequences [18,30,31,32,33,34,35,36,37,38,39,40]. Based on these findings, using CBT or its variants (i.e., mindfulness based cognitive therapy (MBCT) which combines CBT elements with meditation) might be able to modulate the activity of DMN and result in clinical improvement [30]. 

A fourth network seems also to be implicated in the generation of some symptoms of MDD. It is the fronto-striatal network which is responsible for reward processing; a processing that consists of different temporal phases (i.e., selection, anticipation and feedback) during which the individual may differentially engage different components of the devoted network [18,41]. In this network, the medial PFC seems to exert a top-down control over the reward promoting areas such as the ventral tegmental area, nucleus accumbens, and ventral pallidum, whereas the lateral habenula seems to constitute the aversive center involved in encoding negative valence [41]. A hypoconnectivity in the fronto-striatal reward circuitry and/or a hyperactivity in the aversive center might account for anhedonia (i.e., loss of interest, motivation, and pleasure) [18,41]. In this perspective, using CBT or its components (i.e., behavioral activation) might be of help in targeting the reward circuitry and acting on behavioral MDD symptoms.

## 4. Underlying Mechanisms of CBT in Depression: Neuroimaging Data Results

Seventeen studies were identified. Three of them applied CBT variants such as MBCT (*n* = 1 study) and behavioral activation therapy for depression (BATD; *n* = 2 studies). The imaging techniques were functional MRI (fMRI) in eleven studies (*n* = 1 resting-functional connectivity and *n* = 10 during task performance), proton magnetic resonance spectroscopy (^1^H-MRS) in two studies, positron emission tomography (PET) in three studies, and single photon emission computed tomography (SPECT) in one study. Only one study was randomized comparing CBT to pharmacological interventions, and none included an effective placebo control (waiting-list or no-treatment control). Studies are summarized in Table A1.

### 4.1. MRI Studies

#### 4.1.1. fMRI Studies

##### Pattern of Regional Activity

Most of the available fMRI studies were in favor of CBT’s effects on the abovementioned top-down and bottom-up processes involved in cognitive control and generation of emotions, respectively. In one of the earliest studies, Fu and colleagues employed a task aiming to assess implicit processing of sad faces in MDD patients receiving CBT (16 sessions) compared to a control group of healthy subjects [42]. Patients exhibited higher baseline activity within the right AHC than controls. Following CBT, patients exhibited a decrease in right AHC activity; and an increase in ACC extending to the superior frontal gyrus, PCC, inferior parietal cortex, and precuneus. Consistent with this study, Ritchley and colleagues assessed the effects of ~20 CBT sessions in MDD patients compared to an untreated group of healthy controls [43]. Both groups underwent baseline and follow-up fMRI during the performance of a task that assesses neural responses to positive, negative, and neutral pictures. At baseline, compared to controls, patients exhibited a reduced activity in the ventromedial PFC, decreased discrimination between emotional and neutral stimuli within the amygdala, and a negativity bias in valence-related activity within the anterior temporal lobe, all of which were reversed following CBT and were paralleled with symptom improvement.

Same results were also reported by several authors in prefrontal and/or limbic areas. Concerning prefrontal regions, Yoshimura and colleagues studied the effects of 12 CBT sessions in MDD patients compared to an untreated healthy control group by employing fMRI during the performance of a self-referential task using emotional words at baseline and follow-up [44]. Following CBT, patients displayed an increase in the activity of ventral ACC and medial PFC when patients considered that positive words described them. However, in the same study, a post-CBT decrease in the activity of the aforementioned area was observed when patients considered that negative words described them, highlighting the contribution of task choice to study outcome [44]. Yang and colleagues adapted fMRI during a cognitive control task, and documented CBT related improvement paralleled by an increase in regional activities related to cognitive control of emotions (i.e., dorsal ACC, dorsolateral and ventrolateral PFC) [45].

As for the limbic activity, Sankar and colleagues enrolled MDD patients treated with CBT (16 sessions) and healthy controls and applied a fMRI paradigm during statements attribution derived from a modified Dysfunctional Attitudes Scale [46]. Post-CBT, patients were found to have a decrease in the activity of left parahippocampal gyrus, the activity of which was reported to be increased in depression [47,48]. This might reflect a CBT-related improvement in dysfunctional thinking. In the same study, the authors documented a decrease in the activity of the PCC, a key component of the DMN [30].

Of interest, the abovementioned results observed post-CBT are consistent with those obtained following pharmacological interventions for depression, which documented an increase in the activity of prefrontal areas (i.e., dorsolateral, dorsomedial and ventrolateral PFC) and a decrease in the activity of some limbic regions (e.g., AHC) following pharmacological interventions in depression patients [49]. However, ACC activity seems to increase following CBT [42,44,45] and decrease following antidepressant medications [49], suggesting treatment-related specific effects over this cerebral area.

Conversely, two studies obtained opposite fMRI changes following psychotherapeutic interventions. In the first one, Rubin-Falcone and colleagues assessed fMRI changes in CBT-treated patients (14 sessions) and healthy controls during the performance of a task consisting of a voluntary emotion regulation strategy while recalling negative autobiographical memories. The observed clinical improvement was associated with a decrease in the activity of the medial PFC cortex and subgenual ACC [50]. In the second study, Dichter and colleagues assessed the biological mechanisms of BATD, a therapy that focuses on the behavioral activation component of CBT and entails reducing avoidance behaviors and increasing engagement with potentially rewarding behaviors [51]. In line with Rubin-Falcone and colleagues’ results, BATD led to a decrease in prefrontal activity (including the paracingulate gyrus, right OFC, and right frontal pole) during the processing of cognitive control stimuli presented within a sad context [51]. Although inconsistent with previous findings, the results of both studies could be partly attributed to the choice of stimuli (i.e., negative in this study) as seen previously in Yoshimura and colleagues’ study where, in the same cohort, post-CBT decrease and increase in prefrontal activities were observed in response to negative and positive stimuli, respectively [44]. It is noteworthy that Dichter and colleagues also employed fMRI during reward processing and reported BATD related functional improvement in areas that are part of the reward circuit during reward selection (i.e., the paracingulate cortex), reward anticipation (i.e., the dorsal striatum), and reward feedback (the paracingulate cortex and OFC) [52].

A third group of authors, like Siegle and colleagues, failed to document fMRI changes following CBT [53]. These researchers employed fMRI during a task sensitive to sustained emotional information processing (i.e., self-ratings of emotionally valanced words) in a group of MDD patients treated with CBT (16–20 sessions) and untreated healthy controls. They focused on the baseline activity within the subgenual ACC based on previous studies that suggested this area as a modulator (inhibitor) of emotions via its connections with limbic regions (i.e., amygdala) [54,55,56]. Although higher treatment effectiveness was observed among participants who had lower activity in the subgenual ACC prior to CBT which support previous findings, no significant changes in the activity of this structure were observed following CBT. The lack of such effects might be explained by methodological limitations (i.e., potential index’s low reliability), but may also imply that post-CBT clinical improvement was unrelated to changes in subgenual ACC activity. Another issue would be the lack of sufficient sociodemographic and clinical information limiting the possibility to compare the results with those obtained in other studies.

##### Functional Connectivity

Other research teams adapted different imaging modalities to understand CBT’s effects. In a first study, Shou and colleagues assessed the resting-state functional amygdalo-cortical connectivity in CBT-treated patients (12 sessions) and controls [57], based on their previous study that documented hypoconnectivity between these regions in patients with MDD [58]. Following CBT, patients exhibited an enhancement in resting-state functional connectivity between the amygdala and frontal regions that are believed to exert cognitive control. Again, findings of this study support the hypothesis that CBT may increase top-down cognitive control exerted by ‘emotionally cold’ areas (i.e., neocortex) over ‘emotionally hot’ areas (limbic regions).

Furthermore, Yoshimura and colleagues have focused on functional connectivity between medial PFC and ACC (during self-referential judgment of negative adjectives) which was high at baseline and decreased following CBT [59]. Medial PFC and ACC are components of the DMN which was previously found to exhibit a hyperconnectivity pattern among its components in patients with MDD, possibly reflecting aberrant cognitive and affective processing and dysfunctional self-focus [37,60]. Therefore, CBT may have exerted its effects by breaking the increased coupling between medial PFC and ACC. However, these results should be interpreted with caution admitting the major limitations of this study, namely the inadequate control group and the fact that patients were receiving concomitant pharmacological therapy.

#### 4.1.2. MRS Studies

Sanacora and colleagues assessed CBT (12 sessions) mechanisms using ^1^H-MRS in an uncontrolled study involving eight patients with MDD [61]. No significant changes (a trend toward a decrease) were observed with regards to resting state occipital cortex gamma-aminobutyric acid (GABA) following CBT. Here, it is important to note that ^1^H-MRS studies have shown an increase in GABA following treatment with selective serotonin re-uptake inhibitors (SSRIs) and electroconvulsive therapy (ECT) [62,63]. Therefore, CBT may have different mechanisms of action than those seen with pharmacological and ECT interventions. The absence of effects in this study might be also related to the small sample size and needs to be further explored.

In a second study, Li and colleagues, authors applied an eight-week MBCT protocol (8 weekly sessions and 6 days/week homework), in a group of depressed patients compared to an untreated healthy control group [64]. Following CBT, an increase in the N-acetyl aspartate/total creatine (NAA/tCr) ratio—a marker of neuronal function- in the left ACC was observed and was significantly associated with clinical improvement. MBCT seems to enhance the function of ACC, a region involved in cognitive, affective and default mode networks that were reported to be dysfunctional in the context of MDD [18]. Such a finding is in line with pharmacological studies documenting NAA increase within the ACC following antidepressant medications in patients with MDD [65].

### 4.2. PET Studies

Results of PET studies derive from the same team, are inconsistent with the above seen results and partly support the abovementioned data linking CBT with the cognitive model of depression. In the first (nonrandomized) one, Goldapple and colleagues employed resting-state fluorine-18 labeled deoxy-glucose PET in patients with MDD and treated with either CBT (≥12 sessions) or antidepressant medications (i.e., paroxetine) [66]. In the second trial, patients were randomized to receive either CBT (15–20 sessions) or antidepressant medications (i.e., venlafaxine) and underwent similar PET scan evaluations [67]. As is seen in most fMRI studies, a significant increase in ACC activity was noted following CBT. However, unlike the majority of fMRI studies (except [50,51]), PET scan studies revealed a decreased metabolism in frontal regions [66,67] and increased activity in limbic regions (i.e., hippocampus [66]) following CBT. Although these findings are intriguing and may not support the cognitive model discussed earlier, several factors could have contributed to their occurrence. First, these results could be interpreted as a treatment-related decrease of ruminations and maladaptive associative memories. Second, the discrepancy between these outcomes and those observed with fMRI could be due to the differences in the measured outcomes of each technique (fMRI vs. PET), but also to the type of control chosen, that consisted of untreated healthy controls in fMRI studies, and of depressed patients pharmacologically-treated in above-cited PET studies. Interestingly, although both treatments (CBT vs. pharmacological intervention) yielded comparable clinical outcomes and some common changes on PET scan [67], they resulted in overlapping, yet opposing, changes in cerebral areas such as the ACC, hinting toward different underlying mechanisms of actions of these therapies in some brain regions [66,67].

It is worth noting that, in both studies, in addition to CBT effects on the cognitive and affective networks, the authors observed a decrease in the metabolic activity of the inferior parietal cortex [66] and PCC [66,67], both of which are components of the DMN that displays enhanced connectivity in the context of MDD. Therefore, CBT might have acted by deactivating this hyperfunctioning network.

In a third study which was based on the serotonin hypothesis of depression, the authors employed a pharmacologically-based PET scanning (using 5-hydroxytryptamine 1B (5-HT1B), an autoreceptor selective ligand) [68]. Following internet-based CBT (ten modules over 10–12 weeks, open-label design, no control group), Tiger and colleagues reported a significant reduction of radioligand binding potential in the dorsal brain stem (DBS) which accounts for around 85% of all serotoninergic neurons in the brain [69]. This could be interpreted as a downregulation of 5-HT1B receptor messenger RNA, which has been previously observed in rodents chronically treated with antidepressant medications [70]. Such a downregulation may remove the inhibition on DBS serotoninergic neurons and therefore induce an increase in serotonin release from its projections in mood-related brain areas (i.e., prefrontal and limbic regions) [69,71]. However, given the absence of correlation between the PET reported changes and clinical improvement and in the absence of a control group, results should be interpreted with caution. It might be better to consider these PET changes as parallel findings rather than mediators of the clinical response.

### 4.3. SPECT Studies

Amsterdam and colleagues performed a nonrandomized single photon emission computed tomography (SPECT) to assess serotonin transporter (SERT) binding capacity [72]. The authors employed a radiopharmaceutical, the 2-((2-((Dimethylamino)methyl)phenyl)thio)-5-(123)I-iodophenylamine ((123)I-ADAM), which selectively binds to serotonin transporters in the central nervous system [73]. The authors compared a group of depressed patients treated with CBT (16 sessions over twelve weeks) to a group of untreated healthy controls. Patients were found to have low SERT standardized uptake ratios at baseline as seen in some but not all studies assessing depression [74,75,76,77,78]. This baseline result could reflect low brain serotonin levels, a finding that seems to improve in the midbrain, right and left medial temporal lobes following CBT.

## 5. Discussion

Although only few data are available on the biological effects of CBT in patients with depression, there is some evidence regarding CBT-effects on activity or metabolism in specific cortical and subcortical areas, mainly in prefrontal regions, cingulate regions, amygdala and hippocampus (fMRI and PET scan data). However, the direction of changes varied across the studies as will be discussed in this section. Other neuroimaging studies (MRS, SPECT) have suggested a modulation of serotonergic transmission [68,72] or an improvement in neuronal function [65] following CBT.

The main issue that challenges formal comparisons of findings across the studies is the heterogeneity in the cohorts, type of psychotherapy, protocol designs and employed techniques.

To start, the mean age ranged from 30 to 47.8 years [64,68], and female proportion ranged from 30.4 to 87.5% [44,45,59]. Some studies assessed CBT’s impact in patients with first acute depressive episodes, while others investigated the effects of this approach in cohorts with recurrent chronic illness. The majority of trials included patients with moderate depressive symptoms, while two studies included patients with mild depressive symptoms [44,59]. Depression assessment tools varied as well and consisted of the Hamilton depression rating scale (HDRS), Beck depression inventory (BDI) and Montgomery Asberg depression rating scale (MADRS) [79,80,81]. In the majority of studies, patients were drug free at the time of intervention, except in two studies where patients were drug-resistant, treated with one or more antidepressant drugs for a minimum of eight weeks prior to inclusion and continued to receive medications during the study period [44,59].

Second, the protocols also differed in the type of therapy (methodological differences between conventional CBT, MBCT and BATD [82,83,84]), the duration of sessions (ranging from 8 sessions in the MBCT protocol [64] and up to 35 sessions in some CBT protocols [43]), the application milieu (individual versus group therapy), and the mode of delivery (single therapist versus multiple therapists versus internet-based intervention).

Third, regarding protocol design, an ideal study would take the form of a randomized controlled trial that would include several treatment arms: depressed patients receiving CBT, depressed patients receiving active nonpharmacological control intervention (non-CBT therapy), depressed patients receiving antidepressant medications, untreated healthy controls, and healthy controls receiving CBT. However, such a design is very challenging and would raise ethical concerns that would prohibit or discourage including untreated group of patients with acute symptoms. Most of the available studies were nonrandomized or open label trials, with or without a control group of untreated healthy controls. Only one of them was randomized [67] and only two included a treatment control arm which consisted of antidepressant medications [66,67]. Future studies would benefit from implementing an effective control arm to be able to judge whether the observed changes are true CBT’s effects or result from sympathetic patients’ care or spontaneous recovery.

Fourth, the available studies differed in the neuroimaging paradigms; mainly, the employed imaging modalities, the selected regions of interest and the choice of cognitive/emotional task adapted during imaging [85]. For instance, regarding the adopted technique, while fluorine-18 labelled deoxy-glucose and 5-HT1B auto-receptor selective radioligand based PET scans work by measuring glucose metabolism or serotonin distribution respectively, fMRI measures deoxyhemoglobin concentration. In addition, admitting that the employed techniques measure different physiological parameters, and differ in spatial and temporal resolutions, findings could considerably differ among the studies. Moreover, using the same technique (i.e., fMRI), some studies have focused on measuring changes in resting state functional connectivity of brain networks while others have examined changes in task-dependent brain activity pattern post CBT. Furthermore, tasks also varied across studies and might account for the discrepancy in the observed changes. For instance, following CBT, ACC activity (i) decreased during processing of negative stimuli (i.e., negative autobiographical memories or self-referential processing of negative stimuli) [44,50], (ii) increased when acquiring PET scan at rest [66,67] or fMRI during facial emotion processing (i.e., sad faces) [32], self-referential processing of positive stimuli [44], or cognitive control of emotions [45]; (iii) or remained unchanged when adapting a task consisting of self-rating emotionally valanced words [53]. Similar findings were obtained in other brain areas such as the PFC, the activity of which was found to be (i) decreased when adapting resting state PET scan [66,67], or when employing fMRI during the processing of negative stimuli (self-referential processing of negative stimuli or processing of negative autobiographical memories [44,50]); and (ii) increased during when responding to emotional and neutral pictures [43], during self-referential processing of positive stimuli [44], or when engaging in a cognitive control task [45]. The same applies to the AHC activity which was found to be (i) increased (hippocampus) during resting state PET scan [66] or (ii) decreased when adapting fMRI during an affect processing task [42].

## 6. Conclusions

Despite the above-described incongruence in the activity pattern, the identified regions appear to play a key role in the fronto-limbic system involved in emotional experience and control. This suggests that CBT-modulatory effects lie within the previously mentioned dorsal cognitive and ventral limbic networks. Very few data also support CBT effects over DMN [59] or the reward circuit [52]. However, facing the dearth of the available data, future randomized controlled trials are highly needed in order to draw formal conclusion on CBT mechanisms of action in large MDD cohorts.

## Appendix A

**Table A1:** Neuroimaging studies assessing cerebral changes following cognitive behavioral therapy.

Study	Patients Age	Patients Sex	Symptom Severity	Antidepressant Medications	Imaging Technique	Psychotherapy Protocol	Control Group	Outcome
***PET scan and related imaging techniques***								
Goldapple et al., 2004 [66]	41.0 ± 9.0 years	11F/6M (64.7% F)	Baseline HDRS scores: 20.0 ± 3.0 (Moderate depression)	Drug free (*n =* 6 drug naïve; *n =* 1 patient required antidepressant washout 4 weeks prior to inclusion; none was considered treatment refractory)	Fluorine-18-labeled deoxyglucose PET scan at rest	15–20 individualized sessions (17.7 ± 2.0 sessions over 26 ± 7 weeks) according to Beck et al. [7]: -Behavioral activation -Cognitive monitoring -Patients’ testing of their interpretations and beliefs (behavioral experiments) -Patients’ using of thoughts records	13 paroxetine-treated patients	Post-CBT increase in glucose metabolism in hippocampus and ACC activity and decrease in glucose metabolism in dorsal, ventral and medial frontal cortex Post-paroxetine increase in PFC activity and decrease in subgenual cingulate and hippocampus activity
Kennedy et al., 2007 [67]	30.0 ± 9.8 years	7F/5M (58.3% F)	Baseline HDRS score: 22.3 ± 4.2 (Moderate depression)	Drug free for at least 2 weeks (4 weeks for fluoxetine)	Fluorine-18-labeled deoxyglucose PET scan at rest	14.1 ± 3.1 individualized sessions over 16 weeks according to Beck et al. [7]: -Strategies aiming to reduce automatic reactivity to negative thoughts or attitudes and to combat dysphoric mood	12 venlafaxine-treated patients	Post-CBT and post-venlafaxine decrease in glucose metabolism in the OFC bilaterally and left medial PFC, along with an increase in glucose metabolism in the right occipital-temporal cortex Venlafaxine-specific decrease in posterior subgenual cingulate metabolism CBT-specific increase in subgenual cingulate metabolism in areas that are anterior, dorsal or medial to those modified with venlafaxine
Amsterdam et al., 2013 [72]	41.0 ± 12.8 years	5F/15M (25.0% F)	Baseline HDRS score: 20.3 ± 2.5 (Moderate depression)	Drug free for at least 12 months (*n =* 10 drug-naïve; *n =* 10 previously treated with antidepressant medications)	[123I]-ADAM SPECT (SERT radioligand) at rest	16 sessions (50 min each) according to Beck et al. [7]: -Promoting behavioral activation and counter-acting maladaptive cognitive biases -Identification and evaluation of underlying beliefs and schemas -Cognitive-behavioral skills-training including homework	10 HCs	Post-CBT increase in Low SERT binding (standardized uptake ratio) in the midbrain, right medial temporal lobe, and left medial temporal lobe
Tiger et al., 2014 [68]	47.8 ± 16.6 years	6F/4M (60% F)	Baseline MADRS score: 26.0 ± 3.9 (Moderate depression)	Drug free for at least 1 month (*n =* 4 drug-naïve; medication-free period: 5.2 ± 72.9 years)	Serotonin (5-HT1B) receptor selective radioligand [11C]AZ10419369 PET scan at rest	Internet based CBT over 10–12 weeks (11.9 ± 71.4 weeks) according to Andersson et al. [83]: -Introduction-Behavioral activation -Cognitive restructuring -Sleep and physical health -Relapse prevention -Future goals	N/A	Post-CBT decrease in binding potential found in the dorsal brain stem post-CBT suggesting increased serotonin release
***fMRI studies***								
Fu et al., [42] 2008	40.0 ± 9.4 years	13F/3M (81.3% F)	Baseline HDRS score: 20.9 ± 1.9 (Moderate depression) Baseline BDI score: 38.0 ± 11.7 (Severe depression)	Drug free for at least 4 weeks (8 weeks for Fluoxetine)	fMRI during sad facial affect recognition task	16 sessions over 16 weeks according to Beck et al. [7]	16 HCs	Post-CBT normalization of the high baseline hippocampus-amygdala activity
Dichter et al., 2009 [52]	39.0 ± 10.4 years	6F/6M (50.0% F)	Baseline HDRS score: 23.8 ± 2.3 (Moderate depression) Baseline BDI score: 27.1 ± 5.1 (Moderate depression)	Drug free at the time of first neuroimaging	fMRI during a decision-making task that dissociates reward choice selection, anticipation, and feedback	11.4 ± 2.0 weekly sessions of BATD: -Encouraging patients to expose themselves to reinforcing situations and to inhibit the behavioral withdrawal	15 HCs	Post-BATD improvement in regional activity within the reward circuit including the striatum and prefrontal areas (paracingulate gyrus and OFC)
Dichter et al., 2010 [51]	39.0 ± 10.4 years	6F/6M (50.0% F)	Baseline HDRS score: 23.8 ± 2.3 (Moderate depression) Baseline BDI score: 27.1 ± 5.1 (Moderate depression)	Drug free at the time of first neuroimaging	fMRI during a task requiring cognitive control in both sad and neutral contexts.	11.4 ± 2.0 weekly sessions (same as Dichter et al., 2009)	15 HCs	Post-BATD decrease in the prefrontal activity (including the paracingulate gyrus, right OFC, and right frontal pole) during the processing of cognitive control stimuli presented within a sad context
Ritchey et al., 2011 [43]	36.1 ± 10.1 years	8F/3M (72.7% F)	Baseline HRSD score: 26.7 ± 6.7 (Moderate depression) Baseline BDI score: 23.0± 8.7 (Moderate depression)	Drug free for at least 2 months	fMRI during Emotion evaluative task (positive, negative and neutral)	20.7 ± 7.6 sessions (50 minutes each) over 30.3 ± 12.5 weeks according to Beck et al. [82]: -Identifying and challenging negative automatic thoughts and core beliefs -Conducting behavioral experiments	7 HCs	Post-CBT change in ventromedial PFC hypoactivity and amygdala hyperresponsiveness in the direction of normalization
Siegle et al., 2012 [53]	40 patients; age N/A	Details N/A	BDI scores N/A	Drug free for at least 2 weeks (6 weeks for fluoxetine)	fMRI during sustained emotional information processing task	16–20 sessions over 12 weeks according to Beck et al. [7]: -Identifying thought/feeling relationships -Learning skills for challenging negative thoughts and adopting more adaptive thoughts -Homework (cognitive and behavioral exercise)	Details N/A	Absence of post-CBT increase in baseline subgenual ACC hypoactivity
Sankar et al., 2014 [46]	39.9 ± 9.5 years	13F/3M (81.3% F)	Baseline HDRS score: 20.9 ± 1.9 (Moderate depression)	Drug free for at least 4 weeks (8 weeks for fluoxetine)	fMRI during attributions to statements from a modified Dysfunctional Attitudes Scale	16 sessions over 16 weeks according to Beck et al. [7]	16 HCs	Post-CBT decrease in left hippocampal activity
Yoshimura et al., 2014 [44]	37.3 ± 7.2 years	7F/16M (30.4% F)	Baseline HDRS score: 11.0 ± 4.8 (Mild depression) Baseline BDI score: 21.4 ± 8.5 (Moderate depression)	Treatment with one or more antidepressant drugs for a minimum of 8 weeks prior to inclusion (without remission) and during the study period	fMRI during self-referential task using emotional (positive and negative) trait words	12 weekly group sessions according to Beck et al. [7]: -Psychoeducation about depression and CBT, -Understanding the relationship between cognition and mood -Self-monitoring of automatic thoughts, behaviors and mood -Identifying and challenging negative self-talk -Challenging and restructuring negative thinking about the self -Looking for new ideas and invoking positive thinking and practicing them in daily life -Evaluating one’s own ideas and thinking during the last week-Setting up an action plan for the next week -Reviewing the outcome of the program -Relapse prevention	15 HCs	Post-CBT increase in medial PFC and ventral ACC activity when processing positive stimuli Post-CBT decrease in the activity of the same regions when processing negative stimuli
Yoshimura et al., 2017 [59]	37.4 ± 7.1 years	10F/19M (34.5% F)	Baseline HDRS score: 11.5 ± 5.7 (Mild depression) Baseline BDI score: 21.4 ± 8.8 (Moderate depression)	Treatment with one or more antidepressant drugs for a minimum of 8 weeks prior to inclusion (without remission) and during the study period	fMRI during self-referential task using emotional (positive and negative) trait words to assess connectivity between medial PFC and ACC	12 weekly group sessions (90 min each); same as Yoshimura et al., 2014	15 HCs	Post-CBT decrease in functional connectivity between the medial PFC and ACC which are part of the default-mode
Shou et al., 2017 [57]	31.9 ± 6.6 years	Predominantly females (details N/A)	Baseline MADRS score: 28.41 ± 6.1 (Moderate depression)	Drug free for at least three weeks (five weeks for fluoxetine)	Resting-state fMRI assessing functional connectivity of the bilateral amygdala with the fronto-parietal network	12 CBT sessions over 12 weeks according to Beck et al. [7]: -Sessions targeting faulty cognition and emotion regulation	18 HCs	Post-CBT increase in connectivity between the amygdala and the fronto-parietal network
Yang et al., 2018 [45]	34.6 ± 8.3 years	14F/2M (87.5% F)	Baseline MADRS score: 27.4 ± 6.0 (Moderate depression)	Drug free for at least 3 weeks (5 weeks for fluoxetine)	fMRI during cognitive control task: paying attention to either houses or fearful/neutral faces presented in a target axis while ignoring the stimuli presented in the other axis	CBT sessions over 12 weeks	19 HCs	Post-CBT increase in regional activity within the cognitive control network, including ventrolateral and dorsolateral PFC
Rubin-Falcone et al., 2018 [50]	34.2 ± 10.20 years	19F/12M (55% F)	Baseline HDRS score: 19.1 ± 4.4 (Moderate depression) Baseline BDI score: 28.0 ± 7.7 (Moderate depression)	Drug free (*n =* 14 drug naïve; *n =* 15 previously treated with antidepressant drugs; *n =* 2 required antidepressant drugs washout 3 weeks prior to inclusion).	fMRI during engagement in a voluntary emotion regulation strategy while recalling negative autobiographical memories	14 sessions (45 minutes each) over 12 weeks according to Beck et al. [7]: -Cognitive-restructuring -Behavioral activation -Behavioral experiments as a means to examine negative automatic predictions -Identifying and modifying more deeply held patterns of negative thinking about oneself, one’s life, and one’s future	18 HCs	Post-CBT better outcome associated with a decrease in the activity of subgenual ACC and medial PFC
***MRS studies***								
Sanacora et al., 2006 [61]	8 patients; details N/A	Details N/A	Baseline HDRS score: 28.1 ± 8.7 (Moderate depression)	Drug free for at least 2 years (*n =* 1 patient discontinued alprazolam 3 weeks prior to inclusion)	^1^H-MRS to measure occipital GABA concentrations at rest	12 sessions over 12 weeks according to Beck et al. [7]	8 patients receiving ECT 11 patients receiving antidepressant medications	Post-CBT trend toward a decrease in occipital GABA concentration Post-ECT and antidepressants increase in occipital GABA concentration
Li et al., 2016 [64]	30.0 ± 7.0 years	11F/5M (68.8% F)	Baseline HDRS median (range): 18 (13–24) (Moderate depression)	Drug free for at least 6 weeks	^1^H-MRS to estimate metabolite ratios in insula, ACC, caudate, putamen, and thalamus	MBCT according to Segal et al. [84]: -8 weekly 2.25-h classes -45 min of homework 6 days per week	10 HCs	Post-MBCT increase in *N*-acetyl aspartate/total creatine ratio (a marker of neuronal function) in the left ACC

ACC: Anterior cingulate cortex; BATD: Behavioral activation therapy for depression; BDI: Beck depression inventory; CBT: Cognitive behavioral therapy; ECT: Electroconvulsive therapy; F: Females; fMRI: Functional MRI; HCs: healthy controls; HDRS: Hamilton Depression Rating Scale score; M: Males; MADRS: Montgomery Asberg Depression Rating Scale; MBCT: Mindfulness based cognitive therapy; MRS: magnetic resonance spectroscopy; N/A: Not applicable; OFC: orbitofrontal cortex; PET: Positron emission tomography; PFC: Prefrontal cortex; SERT: Serotonin transporter; SPECT: Single photon emission computed tomography. All values are displayed as mean ± SD unless indicated differently.
